# How Robust is the Evidence for Prehabilitation in Cancer Surgery?: A Systematic Review and Fragility Index Analysis

**DOI:** 10.1245/s10434-025-18138-3

**Published:** 2025-08-21

**Authors:** Sarah Cook, Xiaoqiu Liu, Mark Hancock, Michael Solomon, Cherry Koh, Bora Kim, Sascha Karunaratne, Kate Alexander, Daniel Steffens

**Affiliations:** 1 Surgical Outcomes Research Centre (SOuRCe), Royal Prince Alfred Hospital, Sydney, Australia; 2 Faculty of Medicine and Health, Central Clinical School, The University of Sydney, Sydney, Australia; 3 Statistics Division, The George Institute for Global Health, University of New South Wales, Sydney, Australia; 4 Faculty of Medicine, Health and Human Sciences, Macquarie University, Sydney, Australia; 5 The Daffodil Center, The University of Sydney, Joint Venture with Cancer Council, Sydney, Australia; 6 Institute of Academic Surgery (IAS), Royal Prince Alfred Hospital, Sydney, Australia

**Keywords:** Prehabilitation, Surgery, Cancer, Randomized controlled trials, Fragility

## Abstract

**Background:**

The number of randomized controlled trials (RCTs) exploring the effectiveness of prehabilitation on improving postoperative outcomes for cancer surgery is increasing. Fragility index (FI) and reverse fragility index (RFI) represent the minimum number of participants whose status needs to change from an “event” to a “non-event,” thereby the results change from statistically significant to nonsignificant (or vice versa for RFI). Fragility quotient (FQ) allows for the FI or RFI to be standardized to the sample size of the study. This review aims to examine the robustness of prehabilitation RCTs by assessing their FI, RFI, and FQ.

**Materials and Methods:**

The Allied and Complementary Medicine Database (AMED), Cumulative Index of Nursing and Allied Health Literature (CINAHL), Cochrane Central Register of Controlled Trials (CENTRAL), Embase, Medline, and PsycINFO were searched from inception to December 2023. Eligible articles included RCTs, with parallel arm design, evaluating the effectiveness of prehabilitation intervention on the reduction of postoperative complications in selected major oncologic surgeries. FI and RFI were determined using the R fragility package.

**Results:**

After screening 2486 publications, 76 RCTs met inclusion criteria. Most of the included RCTs explored the effectiveness of nutritional prehabilitation (*N* = 38; 50%). A total of 544 postoperative complication outcomes were reported across all 76 studies, with 25 (4.6%) demonstrating a significant effect and 519 (95.4%) demonstrating a nonsignificant effect of prehabilitation. Overall, the median FI and RFI were 1 (range 1–14) and 4 (range 1–13), respectively.

**Conclusions:**

The current evidence on the effectiveness of prehabilitation for major cancer surgeries is fragile. Changing outcomes for four participants in most studies was sufficient to make a nonsignificant finding significant.

**Supplementary Information:**

The online version contains supplementary material available at 10.1245/s10434-025-18138-3.

One in four deaths from noncommunicable diseases globally is attributable to cancer.^1^ Lung cancer is the top global cause of mortality due to cancer, with approximately 2 million deaths in 2022. Liver and colorectal cancer represent the second and third causes of global mortality due to cancer, respectively, causing approximately 1 million deaths each in 2022.^2^ The *Lancet Oncology* Commission on Global Cancer Surgery estimated that in 2015, more than 12 million people diagnosed with cancer worldwide would require surgical treatment.^3^ Surgical oncology is a core pillar of cancer care, providing preventative, diagnostic, curative, supportive, palliative, and reconstructive care.^3^ However, despite the survival benefit these surgical treatments provide, the rates of postoperative morbidity in this population is high.^4,5^ This surgical treatment comprises major operations, including partial or complete resection of one or more organs and surrounding tissue, with the patient undergoing high levels of metabolic stress.^6^ In 2013, Scaife et al. reported that 10–20% of patients suffer perioperative complications following solid organ tumor resection.^7^ However, the published risk of postoperative complications for individual cancer types ranges from 27%^8^ to up to 60%.^9–11^ Postoperative complications present ramifications for both individuals and healthcare systems. There is a strong association between postoperative complications and decreases in short- and long-term survival rates and long-term quality of life.^12,13^ Postoperative complications increase the length of stay (LOS), readmission rates, and overall cost of treatment; thereby increasing healthcare costs and resource utilization. The causes of postoperative complications are believed to be partly related to the patient’s physiological response to surgical stress.^6,14,15^ Therefore, preoperative interventions aimed at reducing this response to surgical stress may reduce postoperative complication rates.

Specific preoperative characteristics impact postoperative complication outcomes. The postoperative risk of complications increases for patients who experience unintentional weight loss of ≥ 5–10%;^16^ 63% of all patients with cancer will experience some weight loss prior to treatment, with some upper gastrointestinal cancers (gastric and esophageal) reporting rates up to 83%.^17–19^ Up to 56% of patients with gastric or esophageal cancers will experience sarcopenia, that is, where skeletal volume and strength is lost due to malnutrition.^20,21^ Neoadjuvant chemotherapy can cause anorexia, further contributing to preoperative weight loss.^22^ Sarcopenia, weight loss, and malnutrition are all strongly correlated with an increased risk of developing major postoperative complications.^17^

Recent evidence from randomized controlled trials (RCTs) shows that prehabilitation programs may improve a patient’s preoperative functional capacity,^23^ nutritional state,^24^ and/or psychological state,^25^ in turn reducing the risk of major postoperative complications.^26^ Specifically, exercise-based prehabilitation has been shown to significantly reduce the risk of postoperative complications by 50%, with relative risk assessment demonstrating that exercise has a protective role against postoperative complications.^27^ Similarly, preoperative oral immunonutrition for patients at nutritional risk provides a significant (40–60%) decrease in postoperative infectious complications, and reduced hospital LOS.^28^ Therefore, there may be potential benefits for patients with cancer to undergo prehabilitation programs before surgery. While the evidence for prehabilitation is evolving, with many trials being published and an increasing number of registered protocols, there is a need to better understand the robustness of this evidence.

The fragility index (FI) and reverse fragility index (RFI) assist in the interpretation of results of RCTs by determining how robust or fragile the results are.^29^ The FI represents the minimum number of patients for whom events would have to change from an event to a non-event in order for the study results to change from statistically significant to nonsignificant. Lower FI scores indicate that the trial conclusions are more sensitive to small changes in outcomes and thus potentially less robust (i.e., highly fragile).^30^ For instance, a FI score of two indicates that changing the outcome for only two patients in the group with fewer postoperative complications would be sufficient to change the trials result from statistically significant to nonsignificant (*p* > 0.05). RFI calculates how many non-event outcomes would have to be event outcomes for the result to be significant. Therefore, determining the fragility of the current prehabilitation evidence would provide valuable information. Fragility quotient (FQ) describes a study’s fragility as a percentage of the study’s sample size, giving an indication of the percentage of events needed to change for statistical interpretation to change. Through the calculation of FQ, the fragility is standardized to the sample size of a trial.^29^ Like FI and RFI, a smaller FQ results in a less robust study outcome.^31,32^

Therefore, this systematic literature review aims to determine the robustness of RCTs, investigating the role of prehabilitation on reducing postoperative complications in adult patients with cancer undergoing surgery. The specific aims are to determine (i) the FI, RFI, and FQ for postoperative complications reported in prehabilitation RCTs and (ii) whether specific factors, including sample size, year of publication, journal impact factor, cancer type, prehabilitation type number of outcomes, loss to follow-up, or risk of bias, would impact the median RFI of prehabilitation RCTs.

## Materials and Methods

This systematic review follows both the Preferred Reporting Items for Systematic Reviews and Meta-Analyses (PRISMA) and Cochrane guidelines.^33,34^ This systematic review is registered with the National institute for Health and Care Research international prospective register of systematic reviews (PROSPERO) register (ID: CRD42024504919).

### Eligibility Criteria

RCTs reporting the effectiveness of prehabilitation on postoperative complications (a dichotomous outcome), which were randomized according to a 1:1 parallel two-arm design, in patients undergoing surgery for thoracic (lung, esophageal), abdominal (gastric, pancreatic, bile duct, liver, periampullary, colon), and pelvic (colorectal, rectal, bladder, cervical, endometrial, ovarian, prostate) cancers were included. These RCTs needed to compare an intervention (prehabilitation) to the standard of care control. Other study designs, along with abstracts and conference publications, were excluded from this review. Translation was attempted for non-English papers.

### Information Sources

A comprehensive search was performed in the following electronic databases from the earliest record to 11 December 2023: MEDLINE, Embase, Cochrane Library, Cumulative Index of Nursing and Allied Health Literature (CINAHL), Allied and Complementary Medicine Database (AMED), and PsycINFO. In addition to the electronic database searches, forward and backwards citation tracking was conducted for additional relevant articles.

### Search Strategy

A highly sensitive search strategy was developed in conjunction with an experienced librarian from the University of Sydney and was used on the basis of the recommendations of the Cochrane Handbook for Systematic Reviews of Interventions for randomized controlled trials, combined with medical subject headings and keywords to identify potential articles. The search included several terms for “randomised controlled trial” AND “neoplasm” AND “postoperative complications” AND “exercise” OR “nutrition” OR “psychological”, AND “preoperative” (Supplementary Tables 1 and 2).

### Selection Process

In the first stage, titles and abstracts were screened for eligibility and clearly irrelevant studies were excluded (S.C. and S.K.). In the second stage, full-text articles were obtained for each potentially eligible study and assessed according to inclusion criteria. Consensus between the two reviewers was used to resolve any disagreement. If consensus could not be reached, a third reviewer (D.S.) was consulted.

### Data Collection Process

Extracted data included publication year, cancer type, sample size, number of patients lost to follow-up, prehabilitation intervention, journal impact factor, and the dichotomous postoperative complication outcome evaluated. Two review authors (S.C. and S.K.) independently extracted the data from the included studies, and disagreements were resolved through discussion and consensus. If consensus could not be reached, a third reviewer was consulted (D.S.). Studies published in duplicate were only included once, but all versions were considered for maximal data extraction.

### Study Risk of Bias Assessment

The risk of bias in included RCTs were assessed using the revised Risk of Bias tool (RoB 2) of the Cochrane Collaboration.^36^ This tool provides structure to assess the risk of bias in a single result from any type of randomized trial. Five domains are included in the RoB 2 tool that cover the types of bias that impact RCTs. These are biases arising from the randomization process, due to deviations from intended interventions or missing data, measurement of the outcome, or in selection of reported result.^35^ For each domain, a series of signaling questions are answered that are algorithmically mapped to proposed risk of bias judgement.^35,36^ Two reviewers (S.C. and S.K.) independently assessed the risk of bias in all included studies, and consensus was used to resolve any disagreement, or it was achieved through arbitration by a third reviewer (D.S.).

### Effect Measures

FI, RFI, and FQ were calculated in R suite using the fragility package by two independent blinded reviewers (S.C. and X.L.).^37^ The fragility package in R is a validated statistical tool that allows for the calculation of fragility-related metrics using Fisher’s exact tests for dichotomous outcomes. It ensures consistency in how changes in event status affect statistical significance and has been used in multiple clinical methodological studies. Any discrepancies were discussed, and consensus was used to resolve any disagreement. For statistically significant results, FI was calculated by changing the status of one patient from non-event to event in the group with the smaller number of events to keep the total patient number consistent. Then, two-sided Fisher’s exact test was performed to assess statistical significance. Each iteration of this process, changing an event and subsequently assessing statistical significance, increases the number of the FI by one (i.e., one calculation is an FI of one). These iterations continued until the *p* value was > 0.05, deeming the result nonsignificant. For statistically nonsignificant results, RFI was calculated. Unlike FI calculation, a non-event was added to the group with the smallest number of non-events and an event was subtracted from that same group to keep the total patient number consistent. Fisher’s exact test was performed to assess statistical significance. Each iteration of this process increases the RFI by one. These iterations continue until the *p*-value was ≤ 0.05. FQ was calculated for each outcome according to the following equation: $FQ=\left(\frac{\text{FI\, or\, RFI}}{\text {sample size}}\right)\times 100$.

The reported RFI of studies were analyzed in subgroups according to study characteristics, including year of publication, analyzed sample size, number of outcomes, impact factor of published journal, cancer type (genitourinary, lower gastrointestinal (GI), lung, upper GI, or mixed), prehabilitation intervention type (exercise, multimodal, nutrition, psychology), and risk of bias. To keep subgroups relatively equal in size where possible, subgroups based on quantitative characteristics were divided above and below the median, as per subgroup analysis methods by McKechnie et al.^30^

## Results

### Study Selection

**Fig. 1:**
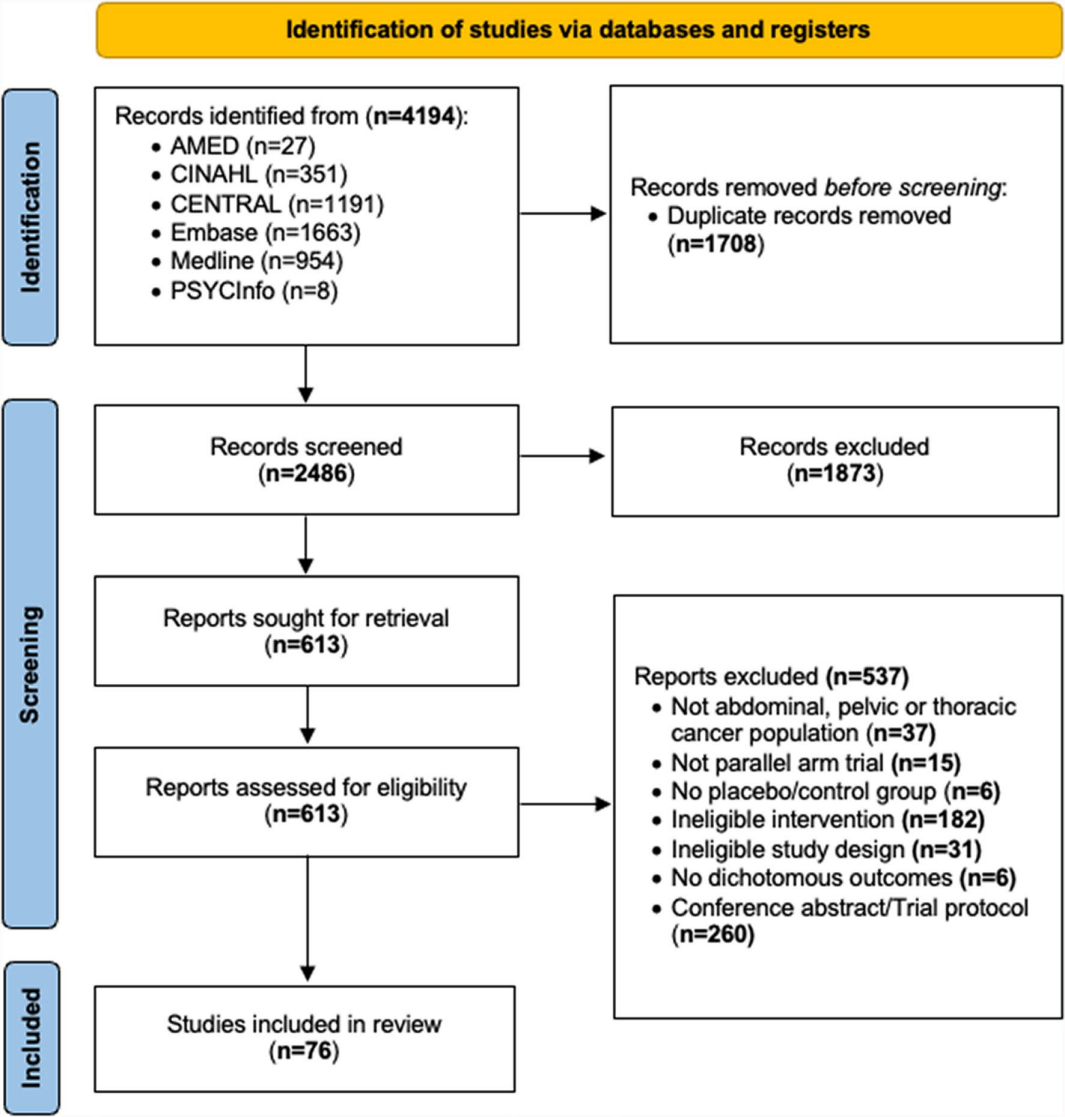
Flow diagram of the included studies

Database searches yielded 4194 records. Prior to screening, 1708 duplicate records were removed, resulting in 2486 records eligible for screening. Through abstract and title screening, 1873 records were excluded. As a result, 613 full-text records were assessed for eligibility. Most exclusions were due to records being conference abstracts or trial protocols (260 reports) or ineligible interventions (182 reports). Thus, a total of 76 RCTs were included (Fig. 1).

### Study Characteristics

A total of 38 (50%) RCTs evaluated nutritional prehabilitation, 27 (36%) evaluated exercise prehabilitation, 10 (13%) evaluated multimodal prehabilitation, and 1 (1%) RCT evaluated psychological prehabilitation (Table 1). In terms of cancer type, 26 (35%) RCTs investigated patients with upper gastrointestinal cancers, 23 (30%) investigated populations with lower gastrointestinal cancers, 15 (20%) investigated populations with lung cancer, 7 (9%) investigated populations with cancer across different organ systems, and 5 (7%) investigated populations with genitourinary cancers. The overall mean sample size was 80 (SD 56.9), and a total of 544 dichotomous postoperative complication outcomes were reported. Of these, 25 (4.6%) outcomes were statistically significant (e.g., *p* < 0.05), and 519 (95.4%) were statistically nonsignificant (e.g., *p* ≥ 0.05).

**Table 1:** Characteristics of included randomized controlled trials (N = 76)

References	Cancer type	Sample size	Lost to follow-up		Intervention details	Impact factor		Postoperative complication outcome	RFI	FQ (%)
*Psychological prehabilitation* (n *= 1*)										
Koet^65^	Lower GI (colorectal)	75 I: 36 C: 39	0		Psychological education (coping strategies and practical, social, and relational problems were addressed); education (colorectal cancer education)	2.8		Complications Clavien–Dindo grade I Clavien–Dindo grade II Clavien–Dindo grade III Clavien–Dindo grade IV	4 6 2 4 4	5.3 8 2.7 5.3 5.3
*Multimodal prehabilitation* (n *= 10*)										
Allen, ^38^	Upper GI (esophageal)	54 I: 26 C: 28	6		Exercise; nutrition; and psychological interventions	3.4		Any complication	3	5.6
Ausania^40^	Upper GI (pancreatic)	40 I: 18 C: 22	0		Aerobic, resistance training, respiratory exercise, and oral nutrition supplementation (including calories and proteins)	2.7		Any complication Major complication Pancreatic leak	3 4 2	7.5 10.0 5.0
Bausys^43^	Upper GI (gastric)	128 I: 61 C: 61	6		Aerobic, resistance training and respiratory exercises, oral nutritional supplement, and psychological interventions	8.6		Pulmonary complications Infections of an unknown source Wound infection Anemia requiring transfusion Anastomotic insufficiency Postoperative bleeding Intraabdominal abscess Pancreatitis or pancreatic fistula Cardiovascular complication Neurological complication Nausea/vomiting Duodenal stump leakage Anastomotic stenosis Urinary tract infection Other	1 1 1 5 3 4 5 5 5 5 3 6 5 5 2*	0.8 0.8 0.8 4.0 2.5 3.3 4.2 4.2 4.2 4.2 2.5 5 4.2 4.2 1.6
Lawson^72^	Lung	34 I: 24 C: 10	12		Exercise, nutrition, and psychological intervention	2.4		30-day complications Clavien–Dindo grade I Clavien–Dindo grade II Clavien–Dindo grade ≥ III	4 4 4 5	14.3 14.3 14.3 17.9
Liu^76^	Lung	85 I: 43 C: 42	12		Aerobic, resistance training, respiratory exercises, oral nutrition supplementation (including protein, whey), and psychological intervention	4.6		Clavien–Dindo grade I Clavien–Dindo grade II Clavien–Dindo grade ≥ III Pneumonia Atelectasis Cardiac complications	4 4 2 4 4 5	5.5 5.5 2.7 5.5 5.5 6.8
Minnella^82^	Upper GI (esophageal)	68 I: 34 C: 34	17		Aerobic, resistance training, dietary advice, and oral nutrition supplementation (including protein, whey)	15.7		Clavien–Dindo grade I Clavien–Dindo grade II Clavien–Dindo grade IIIa Clavien–Dindo grade IVa Clavien–Dindo grade IVb Clavien–Dindo grade V	3 5 2 4 4 4	6.1 10.2 4.1 8.2 8.2 8.2
Minnella^81^	Genitourinary (bladder)	70 I: 35 C: 35	4		Aerobic, resistance training, dietary advice, oral nutrition supplementation (including protein, whey), and psychological intervention 4 weeks	4.8		Clavien–Dindo grade I Clavien–Dindo grade II Clavien–Dindo grade IIIa Clavien–Dindo grade IIIb Clavien–Dindo grade IVa	4 6 3 2 4	6.9 10.3 5.2 3.4 6.9
Molenaar^83^	Lower GI (colorectal)	269 I: 136 C: 133	18		Exercise, nutrition, and psychological support	15.7		Severe complications Overall complications Medical complications	2* 2 2*	0.8 0.8 0.8
Ommundsmen^88^	Lower GI (colorectal)	122 I: 57 C: 65	15		Preoperative geriatric assessment and tailored intervention	2.9		Clavien–Dindo grade I–V Clavien–Dindo grade II–V	2 6	1.7 5.2
Patel^89^	Lung	102 I: 51 C: 51	7		Exercise, nutrition, and psychological intervention	8.6		Incidence of adverse event(s) during hospital admission	4	4.2
*Exercise prehabilitation* (n *= 27*)										
Banerjee^41^	Genitourinary (bladder)	60 I: 30 C:30		5	3–6 weeks high-intensity interval training		2.8	Any complication Respiratory tract infection Major complication	2 4 3	3.6 7.3 5.5
Benzo^44^	Lung	19 I: 10 C: 9		2	Aerobic, resistance training, and respiratory exercises		4.5	Pneumonia Pulmonary complications Respiratory failure	3 2 3	17.6 11.8 17.6
Berkel^45^	Lower GI (colorectal)	74 I: 39 C:35		17	Moderate-to-high intensity aerobic and resistance training 3 weeks		7.5	Any complication Nonsurgical Cardiovascular complication Neurological complication Pulmonary complication Renal complication Thromboembolic complication Other Surgical complication Anastomotic leakage Intraabdominal abscess Sepsis Ileus Abdominal wound complication Urological complication Bleeding Iatrogenic intestinal injury	1* 5 3 4 4 2 5 3 3 5 5 2 4 3 5 5 5	1.7 8.7 5.2 7.0 7.0 3.5 8.7 5.2 5.2 8.7 8.7 3.5 7.0 5.2 8.7 8.7 8.7
Blackwell^46^	Genitourinary (urological)	40 I: 19 C: 21		4	High-intensity interval training		5.1	Any complication Major complication	3 2	7.5 5.0
Christensen^50^	Upper GI (esophageal)	62 I: 27 C: 35		1	High-intensity interval training and resistance training		3.5	All complications Anastomotic leak Serious complications Pneumonia	5 5 5 3	10 10 10 6
Dronkers^51^	Lower GI (colon)	42 I: 22 C: 20		4	Aerobic, resistance training, and respiratory exercises 2–4 weeks		2.6	Any complication Pulmonary Pneumonia	6 5 2	14.6 12.2 4.9
Dunne^52^	Upper GI (liver)	38 I: 20 C: 18		3	High-intensity interval training		8.6	Any complication Clavien–Dindo grades III and IV	5 4	14.7 11.8
Fang^54^	Lung	44 I: 22 C: 22		0	Aerobic and respiratory exercise		0.165	Cardiopulmonary complications Arrhythmia Pulmonary infection Atelectasis Respiratory failure	4 4 3 4 2	9 9 6.8 6.8 4.5
Garcia^98^	Lung	40 I: 20 C: 20		21	Aerobic, resistance training, and respiratory exercise		2.6	Pulmonary complication	3	13.6
Huang^59^	Lung	96 I: 48 C: 48		16	Aerobic, and respiratory exercises		2.1	Pneumonia Pleural effusion Atelectasis Mechanical ventilation > 48 h Acute respiratory distress syndrome Air leak Bronchopleural fistula Clavien–Dindo grade I Clavien–Dindo grade II Clavien–Dindo grade III	1 4 4 5 5 4 5 2 3 5	1.3 5 5 6.3 6.3 5 6.3 2.5 3.8 6.3
Karlsson^61^	Lower GI (colorectal)	23 I: 11 C: 12		2	Aerobic, resistance training and respiratory exercise		2.9	Any complication Respiratory tract infection Wound infection	1 3 3	4.8 14.3 14.3
Kokez^66^	Lung	60 I: 30 C: 30		0	Respiratory exercise training		0.8	Atelectasis Pneumonia Prolonged air leak Arrhythmia Total complication	6 5 2* 5 1	10 8.3 3.3 8.3 1.7
Lai^70^	Lung	48 I: 24 C: 24		0	Preoperative short-term comprehensive pulmonary rehabilitation		1.4	Pulmonary complications Pneumonia Atelectasis Pulmonary embolism Respiratory/heart failure or Air leak	2 5 5 4 4 4	4.16 10.4 10.4 8.33 8.33 8.33
Lai^68^	Lung	60 I: 30 C: 30		0	Aerobic and respiratory exercise. High-intensity exercise including thoracic expansion and incentive spirometry exercises and abdominal breathing and aerobic endurance exercises (Nu-Step device).		1.8	Pulmonary complications Clavien–Dindo grade I New onset purulent sputum Fever > 38 ˚C New rise in CRP or WBC Positive blood cultures Atelectasis Pleural effusion Clavien–Dindo grade II Pneumonia Mechanical ventilation < 48 h Pleural effusion needing tube Atelectasis needing toilet bronchoscopy Clavien–Dindo grade III Empyema Mechanical ventilation > 48 h Chylothorax Clavien–Dindo grade 4 Return to ICU Pulmonary embolism ARDS or respiratory failure Clavien–Dindo grade 5	1 8 6 6 5 5 5 5 4 3 2 6 6 5 3 6 4 5 5 5 5 5	1.7 13.3 10.0 10.0 8.3 8.3 8.3 8.3 6.7 5.0 3.3 10.0 10.0 8.3 5.0 10.0 6.7 8.3 8.3 8.3 8.3 8.3
Lai^69^	Lung	101 I: 51 C: 50		0	Aerobic and respiratory exercise. High-intensity exercise including thoracic expansion and incentive spirometry exercises and abdominal breathing and aerobic endurance exercises (Nu-Step device).		1.6	Pulmonary complications Pneumonia Atelectasis Pulmonary embolism Respiratory/ heart failure or ARDS Mechanical ventilation > 48 h Empyema	2 5 5 4 4 4 5	1.9 4.9 4.9 3.9 3.9 3.9 4.9
Lai^71^	Lung	68 I: 34 C: 34		0	Aerobic and respiratory exercises		3.616	Dyspnea Cough Micro-atelectasis, micro-aerothorax, or air leakage Pneumonia or wound infection Mechanical ventilation < 48 h Pleural effusion needing tube relocation or thoracentesis Atelectasis needing bronchoscope and aspirations Dyspnea pharmacological intervention or ventilator support Air leak Empyema Mechanical ventilation > 48 h Chylothorax Return to ICU Clavien–Dindo grade ≤ I Clavien–Dindo grade II Clavien–Dindo grade III Clavien–Dindo grade IV Pulmonary complication	6 4 6 3 3 6 4 4 5 5 5 5 5 4 2 3 5 1*	8.8 5.9 8.8 4.4 4.4 8.8 5.9 5.9 7.4 7.4 7.4 7.4 7.4 5.9 2.9 4.4 7.4 1.5
Licker^75^	Lung	164 I: 81 C: 83		13	High-intensity interval training		21.0	Respiratory complications ARDS Ventilation (> 6 h) Pneumonia or wound infection Atelectasis Cardiovascular complications Acute coronary syndrome Acute heart failure Pulmonary embolism Stroke Arrhythmias Reoperation Bronchopleural fistula Wound infections Renal dysfunction	3* 4 4 3 7* 5 4 3 4 4 5 1 5 5 4	2 2.6 2.6 2 4.6 3.3 2.6 2 2.6 2.6 3.3 0.6 3.3 3.3 2.6
Mclsaac^79^	Mixed (colorectal, thoracic, hepatobiliary, or urological)	204 I: 102 C: 102		8	Home-based exercise prehabilitation		9.1	Any complication	1	0.5
Mina^97^	Genitourinary (prostate)	86 I: 44 C: 42		25	Aerobic and resistance training		2.3	Clavien–Dindo grade I Clavien–Dindo grade II Clavien–Dindo grade III Clavien–Dindo grade IV	6 4 5 4	7.0 4.7 5.8 4.7
Moug^84^	Lower GI (rectal)	48 I: 24 C: 24		8	Aerobic exercise		2.9	Any complication	4	10.0
Pehlivan^90^	Lung	60 I: 30 C: 30		0	Aerobic and respiratory exercise		1.1	Atelectasis Fever Dyspnea Hemorrhagic drainage Total complications	5 5 5 5 2	8.3 8.3 8.3 8.3 3.3
Peng^91^	Lower GI (colorectal)	213 I:109 C: 104		0	Upper/lower extremity strengthening, thoracic/abdominal breathing exercises, abdominal muscle exercises		7.5	Any complication	5	2.3
Steffens^100^	Mixed (pelvic and abdominal)	22 I: 11 C: 11		0	Aerobic, resistance training, and respiratory exercise		1.5	Clavien–Dindo grade II Clavien–Dindo grade III Clavien–Dindo grade IV Clavien–Dindo grade V Total complications	3 4 3 3 2	13.6 18.2 13.6 13.6 9.1
Swaminathan^101^	Upper GI (gastric)	62 I: 31 C: 31		4	Respiratory prehabilitation. ERAS protocol with respiratory prehabilitation using incentive spirometry		12.5	Clavien–Dindo grade I Clavien–Dindo grade II Clavien–Dindo grade IIIa Clavien–Dindo grade IVa	2 5 5 5	3.4 8.6 8.6 8.6
Valkenet^106^	Upper GI (esophageal)	270 I: 132 C: 138		29	Respiratory exercise		8.6	Death in hospital Pneumonia Antibiotics for suspected pneumonia Pulmonary, other Cardiac Complications, other Anastomotic leak Positive sputum culture Chyle leak Vocal cord paresis Infection, other Sepsis Infection, wound Neurological Delirium/confusion Bleeding Thromboembolic	3 11 8 13 9 3 9 7 7 2 7 5 4 4 1 3 4	1.2 4.6 3.3 5.4 3.7 1.2 3.7 2.9 2.9 0.8 2.9 2.1 1.7 1.7 0.4 1.2 1.7
Xia^108^	Genitourinary (cervical, endometrial, ovarian)	156 I: 78 C: 78		0	Preoperative walking. Low–moderate intensity walking exercise 1 week		0.4	Intestinal obstruction Deep venous thrombosis Incision infection Abdominal infection	14* 4 6 4	9.0 2.6 3.8 2.6
Yamana^110^	Upper GI (esophageal)	63 I: 32 C: 31		3	Aerobic, resistance training, and respiratory exercise		1.8	Clavien–Dindo grade I Clavien–Dindo grade II Clavien–Dindo grade IIIa Clavien–Dindo grade IIIb Anastomotic leakage Chylothorax Recurrent nerve palsy	2* 1* 4 2 3 6 7	3.3 1.7 6.7 3.3 5 10 11.7
*Nutritional prehabilitation* (n *= 38*)										
Ashida^39^	Upper GI (pancreatic)	24 I: 12 C: 12		4	Oral immunonutrition (including calories and protein) and dietary advice		1.8	Overall morbidity Clavien–Dindo grade I Clavien–Dindo grade II Clavien–Dindo grade III Infectious complications Pancreatic fistula Superficial incisional surgical site infection Systemic inflammatory response syndrome	3 1 4 4 2 4 2 4	15.0 5.0 20.0 20.0 10.0 20.0 10.0 20.0
Barth^42^	Upper GI (liver)	63 I: 31 C: 32		3	Oral nutrition supplementation (very low-calorie diet, Optifast 800)		7.5	Any complication	5	8.3
Braga^47^	Upper GI (pancreatic or periampullary cancer)	36 I: 18 C: 18		9	Oral nutrition supplementation (enriched with glutamine, antioxidants, and green tea extract)		3.2	Overall morbidity rate	5	13.9
Burden^49^	Lower GI (colorectal)	125 I: 59 C: 66		5	Oral nutrition supplementation (including calories and proteins) and dietary advice		2.9	Buzby definitions Noninfectious and infectious Wound infection Chest infection Urinary tract infection Total patients with an infection CDC definitions Wound infection Chest infection Urinary tract infection Total patients with an infection	9 3 5 4 8 3 4 4 7	7.7 2.5 4.3 3.4 6.8 2.5 3.4 3.4 6
Burden^48^	Lower GI (colorectal)	101 I: 55 C: 46		20	Oral nutrition supplementation (Fortisip Compact: 10.1 KJ and 0.096 g protein per mL) and dietary advice 5-day minimum		9.4	Clavien–Dindo grade I–II Clavien–Dindo grade III–IVb Surgical site infection Chest infection Urinary tract infection	8 5 1 4 3	8.0 5.0 1.0 4.0 3.0
Fan^53^	Upper GI (esophageal)	40 I: 20 C: 20		0	Parenteral nutrition (including calories and proteins), oral nutrition supplementation (high-energy high-protein diet)		6.6	≥ 1 Postoperative complications Infection Failure Mortality Clinical Subclinical Wound infection Intraperitoneal abscess Intrapleural sepsis Septicemia	3 6 5 5 3 4 3 4 4 4	7.5 15 12.5 12.5 7.5 10 7.5 10 10 10
Fujitani^55^	Upper GI (gastric)	244 I: 127 C: 117		13	Oral immunonutrition (including calories and proteins)		8.6	Surgical site infections Superficial incisional Deep incisional Organ or space Infectious complication Any complication Abdominal abscess Pancreatic fistula Anastomotic leakage Wound infection or discharge Drain infection Pneumonia Venous catheter infection Pleural effusion Postoperative bleeding Ileus Systemic inflammatory response syndrome	10 6 2 9 11 8 5 6 5 4 4 1* 5 5 3 5 6	4.3 2.5 0.8 3.8 4.7 3.4 2.1 2.5 2.1 1.7 1.7 0.4 2.1 2.1 1.2 2.1 2.5
Gade^56^	Upper GI (pancreatic)	46 I: 25 C: 21		11	Preoperative oral immunonutrition		2.0	Septic shock Sepsis Systemic inflammatory response syndrome Anastomotic leak Intraabdominal abscess Cholangitis Pneumonia Local wound infection Fungal infection Infectious diarrhea Vascular insufficiency Cardiac insufficiency Arrhythmia Hypovolemia Multiorgan dysfunction syndrome Renal insufficiency Ileus Chylous Fistula Abdominal bleeding Bleeding from cicatrice Respiratory insufficiency Atelectasis Transient ischemic attack Venous thrombosis Noninfectious wound complication Noninfectious diarrhea Anemia ≤ 2 complications ≥ 3complications ≥ 5 complications ≥ 7 complications	1 4 3 4 4 3 1 1 4 2 1 4 1 1 3 3 4 4 3 2 3 4 3 3 4 3 4 4 1* 1* 2 2	2.9 11.4 8.6 11.4 11.4 8.6 2.9 2.9 11.4 5.7 2.9 11.4 2.9 2.9 8.6 8.6 11.4 11.4 8.6 5.7 8.6 11.4 8.6 8.6 11.4 8.6 11.4 11.4 2.9 2.9 5.7 5.7
Giger-Pabst^57^	Mixed (esophageal, gastric, pancreas, liver, colon, rectum)	108 I: 55 C: 53		5	Oral immunonutrition (impact RTD contains 16.72 g of arginine, 3.3 g of omega-3 fatty acids, and 1.32 g of RNA with a kilocalorie-to-milliliter ratio of 1.4:1)		3.2	Infectious complications Any complications Noninfectious complications Sterile pancreatic fistula Presacral sterile hematoma Heart failure Myocardial infarction Pulmonary embolism Pleural effusion Transient renal failure Anastomotic rectal bleeding Delayed gastric emptying Anastomotic insufficiency Aspiration/ARDS	6 9 7 5 4 5 5 4 4 4 4 5 5 4	5.6 8.3 6.5 4.6 3.7 4.6 4.6 3.7 3.7 3.7 3.7 4.6 4.6 3.7
Hamamoto^58^	Lower GI (colon)	70 I: 35 C: 35		6	Oral nutrition supplementation (carbohydrate loading) 1 day		2.4	Surgical site infection Ileus Leakage	3 2 4	4.7 3.1 6.2
Kabata^60^	Mixed (gastric, colorectal, ovarian, esophageal, liver, appendix)	102 I: 54 C: 48		0	Hypercaloric oral nutrition supplementation (including calories and proteins)		2.8	Wound infection Pneumonia Sepsis Subileus Mechanical ileus Gastric bleeding Esophageal graft perforation Anastomotic leakage Evisceration Fluid-electrolyte disturbances Heart attack Death Total Clavien–Dindo grade I Clavien–Dindo grade II Clavien–Dindo grade III Clavien–Dindo grade IV Clavien–Dindo grade V	1* 5 5 4 4 5 5 2 2 3 3 3 2* 2 4 3 1 3	0.9 4.9 4.9 3.9 3.9 4.9 4.9 1.9 1.9 2.9 2.9 2.9 1.9 1.9 3.9 2.9 0.9 2.9
Kaya^62^	Lung	58 I: 31 C: 27		0	Oral immunonutrition (including calories, proteins, arginine, omega-3 fatty acids, and nucleotides)		1.5	Postoperative air leak Atelectasis Pneumonia Arrhythmia Total	3 2 4 3 1	5.1 3.4 6.9 5.1 1.7
Kikuchi^63^	Upper GI (liver)	77 I: 39 C: 38		0	Oral nutrition supplementation (including branched-chain amino acids)		3.4	Morbidity Clavien–Dindo grade I Clavien–Dindo grade II Clavien–Dindo grade IIIa Clavien–Dindo grade IVa Clavien–Dindo grade IVb Clavien–Dindo grade V	4 4 2 3 5 4 5	5.2 5.2 2.6 3.9 6.5 5.2 6.5
Kitagawa^64^	Upper GI (esophageal)	30 I: 15 C: 15		1	Oral immunonutrition (including calories, proteins, palatinose, and whey)		2.1	Complications Recurrent nerve palsy Infectious complications Pneumonia leak Surgical site infection Clavien–Dindo grade III or IV	4 4 2 3 2 3	13.8 13.8 6.9 10.3 6.9 10.3
Kotzampassi^67^	Lower GI (colorectal)	168 I: 86 C: 82		4	Nutrition: oral probiotic supplementation		2.3	Any major complication Any infectious complication Pneumonia Urinary tract infection Bacteremia Severe sepsis Anastomotic leakage Need for mechanical ventilation	4* 3* 1 2* 4 5 2 1*	2.4 1.8 6.1 1.2 2.4 3 1.2 0.6
Lee^73^	Lower GI (colon)	176 I: 88 C: 88		15	400mL/day immune-nutrient enriched oral nutrition supplementation. Contains high protein, arginine, and omega-3 fatty acids. 7 days		7.5	Total complications Infectious complications Wound infection Organ-space surgical site infection Urinary tract infection Clostridium difficile infection Pneumonia or wound infection Noninfectious Prolonged postoperative ileus Postoperative urinary retention Cardiovascular complication Delirium	10 8 5 2 5 5 4 7 4 5 5 5	6.2 5 3.1 1.2 3.1 3.1 2.5 4.3 2.5 3.1 3.1 3.1
Li^74^	Upper GI (esophageal cancer)	112 I: 56 C: 56		5	Oral immunonutrition		1.9	Clavien–Dindo grade II Clavien–Dindo grade III Clavien–Dindo grade IV Infectious complications Pneumonia Surgical site infection Anastomotic leakage Adverse events Bloating Diarrhea Vomiting	6 5 4 5 5 4 4 9 7 4 4	5.8 4.9 3.9 4.9 4.9 3.9 3.9 8.7 6.8 3.9 3.9
Manzanarez^77^	Lower GI (colorectal)	84 I: 42 C: 42		0	Oral immunonutrition (including calories and proteins)		0.5	Infectious complications Minor complications Major complications	7 3 5	8.3 3.6 6.0
Martin^78^	Upper GI (pancreatic)	71 I: 44 C: 27		0	Oral immunonutrition (including calories and proteins)		3.5	Any complication Cholecystitis Ileus Delayed gastric emptying Pancreatic leak bile leakage Gastritis *C. difficile* colitis Small bowel leak Non-occlusive portal vein thrombosis Elevated bilirubin Ascites Wound infection RUQ fluid Wound seroma Infection of incisional wound or effusion	2 3 1 4 2 2 5 2 3 5 2 2 1 3 3 2	2.8 4.2 1.4 5.6 2.8 2.8 7 2.8 4.2 7 2.8 2.8 1.4 4.2 4.2 2.8
Mikagi^80^	Upper GI (liver)	41 I: 25 C: 16		14	Oral immunonutrition (including calories and proteins)		0.2	Patients with complications Urinary tract infection Ileus Atelectasis	3 4 4 3	11.5 15.4 15.4 11.5
Mueller^85^	Mixed (esophageal, gastric, colorectal, pancreas).	160 I: 80 C: 80		30	Parenteral nutrition (including calories, proteins, and lipids)		98.4	Pneumonia Major complication Wound Infection Mortality	6 1 7 1*	4.8 0.08 5.6 0.8
Nakamura^86^	Upper GI (bile duct, pancreas, gastric, esophageal)	26 I: 12 C: 14		0	Oral immunonutrition (including calories, proteins)		3.2	Cholangitis Bleeding	4 4	15.4 15.4
Okatmoto^87^	Upper GI (gastric)	60 I: 30 C: 30		0	Oral immunonutrition (supplemented with arginine, RNA, and omega 3)		2.3	Infectious complications Infection of incisional wound or effusion Respiratory tract infection Abdominal cavity empyema or effusion Catheter infection Patients with noninfectious complications Cardiac dysfunction Intestinal obstruction Edematous of anastomosis Bleeding	1 5 5 3 5 6 5 5 5 5	1.7 8.3 8.3 5 8.3 10 8.3 8.3 8.3 8.3
Pexe-Machado^92^	Mixed (gastric and colorectal)	30 I: 15 C: 15		18	Oral nutrition supplementation (carbohydrate loading)		3.2	Pneumonia Sepsis Wound infection Ileus Atrial fibrillation	4 4 4 3 3	18.2 18.2 18.2 13.6 13.6
Polakowski^93^	Lower GI (colorectal)	73 I: 36 C: 37		0	Oral nutrition supplementation (probiotic)		3.2	Infections complications Noninfectious complications	1 2	1.4 2.7
Reis^94^	Lower GI (colorectal)	33 I: 15 C: 18		0	Oral nutrition supplementation (carbohydrate loading)		1.7	Clavien–Dindo grade IV–V Reoperation Sepsis Morbidity up to 30 days Intestinal fistula Surgical site infectious	2 4 2 1 3 3	6.1 12.1 6.1 3.0 9.1 9.1
Rizvanovic^95^	Lower GI (colorectal)	60 I: 30 C: 30		0	Carbohydrate loading; 400 mL of a carbohydrate solution at 22:00 h the night before surgery and 200 mL of the same solution 2 h before surgery.		1.7	Elevated serum creatinine Confusion Pneumonia Delirium Urological infection Wound infection Deep vein thrombosis Anastomotic leakage Ileus Abdominal wall dehiscence Reoperation Respiratory failure Renal failure Septic shock Multiorgan failure Death of patient	1 4 1 2 5 4 5 5 5 5 4 4 5 5 5 5	1.7 6.7 1.7 3.3 8.3 6.7 8.3 8.3 8.3 8.3 6.7 6.7 8.3 8.3 8.3 8.3
Russell^96^	Upper GI (liver)	34 I: 17 C: 17		2	Oral immunonutrition (including arginine, omega-3 fatty acids, calories, and proteins)		2.5	Urinary tract Surgical site Blood stream Gastrointestinal Lower respiratory tract AF/bradycardia/tachycardia Acute kidney injury Aspiration pneumonia Acute respiratory distress syndrome Atelectasis Bowel obstruction Diarrhea Electrolyte derangement Encephalopathy Hypotension Ileus Ischemic optic neuropathy Leak Nausea and vomiting Pleural effusion Pain requiring epidural Pneumothorax Noninfected collection Wound dehiscence Patients with an infectious complication Patients with a noninfectious complication Patients with any complication Clavien–Dindo grade ≥ III Clavien–Dindo grade < III	2 4 1* 4 4 4 2 4 4 5 2 4 4 4 3 3 4 4 3 3 3 4 4 4 1 4 4 1* 1	6.3 12.5 3.1 12.5 12.5 12.5 6.3 12.5 12.5 15.6 6.3 12.5 12.5 12.5 9.4 9.4 12.5 12.5 9.4 9.4 9.4 12.5 12.5 12.5 3.1 12.5 12.5 3.1 3.1
Seguin^99^	Upper GI (liver)	35 I: 18 C: 1		0	Oral immunonutrition (including arginine, omega-3 fatty acids, calories, and proteins)		2.0	Abdominal wall abscess Intraabdominal abscess Ascites infection Urinary tract infections Staphylococcal bacteremia *C. difficile*-associated diarrhea Postoperative peritonitis Infectious complications Surgical site infections	3 3 3 2 3 3 3 1 3	8.6 8.6 8.6 5.7 8.6 8.6 8.6 2.9 8.6
Tan^102^	Lower GI (colorectal)	40 I: 20 C: 20		0	Oral nutritional supplement (oral microbial cell preparation)		2.3	Anastomotic leak (grade > 2) Anastomotic leak (grade < 2) Total complications	3 3 3	7.5 7.5 7.5
Tesar^103^	Lower GI (colorectal)	123 I: 62 C: 61		3	Oral nutritional supplements		0.7	Clavien–Dindo grade I Clavien–Dindo grade II Clavien–Dindo grade III Clavien–Dindo grade IV Clavien–Dindo grade V	4 3 5 5 5	3.3 2.5 4.2 4.2 4.2
Tumas^104^	Upper GI (pancreatic)	92 I: 40 C: 52		22	Oral immunonutrition (including l-arginine)		6.6	Complications	2	2.9
Uno^105^	Upper GI (liver)	40 I: 20 C: 20		0	Oral immunonutrition (including calories and proteins)		3.2	Infectious complications Wound infection Intraabdominal abscess Pneumonia Sepsis Noninfectious complications Liver failure (grade B/C) Bile leakage Portal vein thrombosis Others Clavien–Dindo grade I Clavien–Dindo grade II Clavien–Dindo grade III Clavien–Dindo grade IV	1 3 2 3 5 4 2 4 4 5 4 4 1 4	2.5 7.5 5.0 7.5 12.5 10.0 5.0 10.0 10.0 12.5 10.0 10.0 2.5 10.0
Wierdak^107^	Lower GI (colorectal)	30 I: 15 C: 15		3	Oral immunonutrition (including calories and proteins)		4.5	Morbidity	4	15.4
Xu^109^	Mixed (gastric and colorectal)	60 I: 30 C: 30		0	Oral immunonutrition (including calories and proteins)		2.3	Total cases with complication Infection of incisional wound or effusion Respiratory tract infection Urinary tract infection Abdominal cavity empyema of effusion Stoma fistula Postoperative cardiorespiratory dysfunction Postoperative intestinal obstruction	1 4 5 4 5 5 5 6	1.7 6.7 8.3 6.7 8.3 8.3 8.3 10
Yoon^111^	Lower GI (rectal)	40 I: 20 C: 20		4	Oral probiotic supplement (lactobacillus plantarum)		2.9	Clavien–Dindo grade I Clavien–Dindo grade III	2 4	5.0 10.0
Zelic^112^	Lower GI (colorectal)	40 I: 20 C: 20		0	Oral nutrition supplementation (carbohydrate loading)		0.792	Morbidity	4	10.0
Zhang^113^	Lower GI (colorectal)	60 I: 30 C: 30		0	Probiotic treatment; three oral bifid triple viable capsules, each containing 0.21 g of B Longum, L acidophilus and Enterococcus faecalis		2.3	Bacteremia Septicemia Postprocedural pneumonias Intraabdominal abscesses Surgical site infections Perineal infection Anastomotic leak	1* 1* 3 5 3 5 4	1.7 1.7 5.0 8.3 5.0 8.3 6.7

^*^Indicates FI calculations instead of RFI calculations; *I* intervention group, *C* control group

### Risk of Bias

Overall, 49 studies were deemed at high risk of bias (67%). The included studies presented the lowest risk of bias in the following domains: missing outcome data (*N* = 53, 69%), measurement of the outcome (*N* = 52, 68%), and randomization process (*N* = 43, 56%). In total, 59 (77%) studies presented some risk of bias in selection of the reported result (Table 2).

**Table 2:** Risk of bias summary of the included prehabilitation randomized controlled trials (N = 76)

References	Randomization process	Deviations from the intended interventions	Missing outcome data	Measurement of the outcome	Selection of the reported result	Overall
Allen^38^	Low	Some	Low	Low	Some	Some
Ashida^39^	Some	Low	Low	Low	Some	Some
Ausania^40^	Some	High	Low	Low	Some	High
Banerjee^41^	Low	High	Low	Low	Some	High
Barth^42^	Low	High	Low	Low	Some	High
Bausys^43^	Low	Some	Low	Some	Some	Some
Benzo^44^	Some	Some	High	Low	Some	High
Berkel^45^	Low	Low	High	Low	Low	High
Blackwell^46^	Low	High	Low	Low	Some	High
Braga^47^	Low	Low	Low	Low	Some	Some
Burden^49^	Low	Low	Low	Low	Some	Some
Burden^48^	Low	Low	Low	Low	Some	Some
Christensen^50^	High	High	High	Low	Some	High
Dronkers^51^	Low	Low	High	Low	Some	High
Dunne^52^	Low	Low	High	Low	Some	High
Fan^53^	Low	Low	Low	High	Some	High
Fang^54^	Some	Low	Low	Low	Some	High
Fujitani^55^	Low	High	High	Low	Some	High
Gade^56^	Low	Low	Low	Low	Some	Some
Garcia^98^	Low	High	High	Low	Some	High
Giger-Pabst^57^	Low	High	Low	Low	Some	High
Hamamoto^58^	High	Some	Low	Low	Low	High
Huang^59^	Low	Low	High	Low	Low	High
Kabata^60^	Low	Low	Low	High	Low	High
Karlsson^61^	High	Low	Low	Low	Low	High
Kaya^62^	Some	High	High	High	Some	High
Kikuchi^63^	Some	Low	Low	High	Some	High
Kitagawa^64^	Low	Some	High	Some	Some	High
Koet^65^	Some	Low	Low	Some	Some	Some
Kokez^66^	Some	Some	High	High	Some	High
Kotzampassi^67^	Low	Low	Low	Low	Low	Low
Lai^70^	Some	Low	Low	Some	Some	Some
Lai^68^	Some	Some	Low	Some	Some	Some
Lai^69^	Some	Low	Low	Some	Low	Some
Lai^71^	Some	Some	Low	Low	Low	High
Lawson^72^	Some	Some	Some	Some	Some	High
Lee^73^	Low	Some	Low	Low	Low	Some
Li^74^	Low	Low	Some	Low	Some	Some
Licker^75^	Some	Low	Low	Low	Some	Some
Liu^76^	Low	Low	Low	Low	Low	Low
Manzanares^77^	Some	High	Low	Some	Some	High
Martin^78^	Some	High	Low	Some	Some	High
Mclsaac^79^	Low	Some	Low	Some	Low	Some
Mikagi^80^	Some	High	Low	Low	Some	High
Mina^97^	Some	High	Low	Low	Low	High
Minnella^82^	Low	Some	High	Low	Some	High
Minnella^81^	Low	Low	Low	Low	Some	Some
Molenaar^83^	Low	Some	Low	Low	Low	Some
Moug^84^	Some	Some	High	Low	Some	High
Mueller^85^	Some	High	High	High	Some	High
Nakamura^86^	Some	Some	Low	Low	Some	Some
Okamoto^87^	Some	High	Low	High	Some	High
Ommundsmen^88^	Some	Some	Low	Low	Some	Some
Patel^89^	Low	Some	Low	Low	Some	Some
Pehlivan^90^	Some	High	High	Low	Some	High
Peng^91^	Low	Some	Low	Low	Some	Some
Pexe-Machado^92^	Some	High	High	Some	Some	High
Polakowski^93^	Low	High	Low	Low	Some	High
Reis^94^	Some	Some	Low	Low	Some	Some
Rizvanovic^95^	Low	Some	Low	Low	Some	Some
Russell^96^	Low	Some	Low	High	Some	High
Seguin^99^	Low	Low	High	Low	Some	High
Steffens^100^	Low	Some	Low	Low	Some	High
Swaminathan^101^	Low	Some	High	High	Low	High
Tan^102^	Low	Low	Low	Low	Low	Low
Tesar^103^	Low	Some	Low	Low	Some	Some
Tumas^104^	Low	Some	High	High	Low	High
Uno^105^	Low	Some	Low	High	Low	High
Valkenet^106^	Some	Low	High	Low	Some	High
Wierdak^107^	Some	High	Low	Low	Some	High
Xia^108^	Low	Some	Low	High	Some	High
Xu^109^	Some	High	Low	High	Some	High
Yamana^110^	Low	Some	Low	Low	Some	Some
Yoon^111^	Low	Low	High	Low	Low	High
Zelic^112^	Low	High	Low	Low	Some	High
Zhang^113^	Some	High	Low	Low	Some	High

*low* low risk of bias in assessed domain, *some* some concerns of risk of bias in assessed domain, *high* high risk of bias in assessed domain

### Fragility Index and Reverse Fragility Index

FI was calculated for 25 out of the 544 outcomes (4.6%), where the effect of prehabilitation was statistically significant, resulting in a median score of 1 (range 1–14). The calculated FI was less than or equal to the total number of patients lost to follow-up across 18 of 25 outcomes (72%). FQ values for FI calculations ranged from 0.4% to 9%, with a median of 1.7%. RFI was calculated for 519 of the 544 outcomes (95.4%), where the effect of prehabilitation was not statistically significant. The median RFI score was 4 (range 1–13). The calculated RFI was less than or equal to the total number of patients lost to follow-up across 235 of 519 outcomes (56%). FQ values for RFI calculations ranged from 0.08% to 20%, with a median of 6% (Table 1).

### Subgroup Analysis

We conducted an exploratory examination of RFI scores across different study characteristics (Table 3). In this examination, 19 of the 21 subgroups had a median RFI of 4 (91%). Studies with a sample size ≤ 60 or those focusing on mixed cancer types had a median RFI of 3. A median RFI of 4 was observed in the remaining subgroups, which were categorized by publication year, loss to follow-up, number of outcomes, journal impact factor, prehabilitation type, risk of bias, other cancer types, and RCTs with sample sizes > 60. The median FQ values showed variations across these subgroups: RCTs published before 2018 had a median FQ of 8.3% while those published after 2018 had a median FQ of 5.9%. RCTs with < 5% loss to follow-up had a median FQ of 6.3%, while those with ≥ 5% loss to follow-up had a median FQ of 5.6%. RCTs with ≤ 5 reported outcomes showed a median FQ of 7.5%, while those with > 5 outcomes showed a median FQ of 6.7%. For journals with an impact factor < 2.9, the median FQ was 7.6%, while those with an impact factor > 2.9 had a median FQ of 5.6%. Among prehabilitation types, nutritional interventions had a median FQ of 7.5%, while exercise prehabilitation showed a median FQ of 7.3%. Studies with a high risk of bias had a median FQ of 8.3%.

**Table 3:** Subgroup analysis of all included trials (N = 76)

Characteristic (n=number of studies)	RFI, median (IQR)	FQ, median % (IQR)
Year of publication		
< 2018 (n = 37)	4 (2.0)	8.3 (5.5)
≥ 2018 (n = 39)	4 (2.0)	5.9 (5.2)
Sample size		
≤ 60 (n = 38)	3 (1.0)	9.3 (6.1)
> 60 (n = 38)	4 (2.0)	4.2 (2.7)
Loss to follow-up		
< 5% (n = 41)	4 (2.0)	6.3 (4.3)
≥ 5% (n = 35)	4 (1.0)	5.6 (6.8)
Number of outcomes		
≤ 5 (n = 41)	4 (2.0)	7.5 (8.9)
> 5 (n = 35)	4 (2.0)	6.7 (4.9)
Journal impact factor < 2.9 (n = 37) ≥ 2.9 (n = 39)	4 (2.0) 4 (2.0)	7.6 (4.2) 5.6 (7.0)
Cancer type		
Genitourinary (n = 5)	4 (1.5)	5.5 (2.7)
Lower GI (n = 22)	4 (2.0)	5.7 (6.9)
Lung (n = 15)	4 (1.0)	6.8 (4.1)
Mixed (n = 8)	3 (2.0)	4.8 (10.7)
Upper GI (n = 26)	4 (2.0)	8.5 (5.1)
Prehabilitation modality		
Exercise (n = 28)	4 (2.0)	7.3 (5.3)
Multimodal (n = 10)	4 (0.5)	5.5 (3.7)
Nutrition (n = 38)	4 (2.0)	7.5 (6.6)
Psychology (n = 1)^a^	4 (NA)	5.3 (NA)
Risk of bias		
High (n = 48)	4 (2.0)	8.3 (5.1)
Low/some (n = 28)	4 (2.0)	5.1 (4.7)

FQ = $\frac{\text{RFI}}{\text{sample size}} \times 100;$ IQR interquartile range

^a^Unable to calculate median and IQR

Table 4 presents a descriptive subgroup analysis of RFIs from RCTs that listed postoperative complications as a primary outcome. In this analysis, 17 of the 20 subgroups examined had a median RFI of 4 (85%). A median RFI of 3 was observed in RCTs with sample sizes of ≤ 75 or those with ≤ 8 reported outcomes. RCTs investigating mixed cancer types had a median RFI of 5. A median RFI of 4 was observed in the remaining subgroups, which were categorized by publication year, loss to follow-up, journal impact factor, prehabilitation type, risk of bias, other cancer types, RCTs with sample sizes > 75, and RCTs reporting > 8 outcomes. While some subgroups shared the same median RFI, their median FQ values showed numerical differences. These observations are descriptive in nature and were not tested for statistical significance.

**Table 4:** Subgroup analysis of trials with postoperative complications as primary outcome (N = 36)

Characteristic (n = number of studies)	RFI, median (IQR)	FQ, median % (IQR)
Year of publication		
< 2017 (n = 16)	4 (1.5)	5.1 (5.2)
> 2017 (n = 20)	4 (1.0)	4.6 (4.9)
Sample size		
≤ 75 (n = 18)	3 (1.0)	8.3 (3.3)
> 75 (n = 16)	4 (1.0)	3.2 (1.6)
Loss to follow-up		
< 4% (n = 18)	4 (2.0)	6.0 (4.9)
≥ 4% (n = 18)	4 (2.0)	4.0 (3.7)
Number of outcomes		
≤ 8 (n = 20)	3 (1.0)	5.6 (5)
> 8 (n = 16)	4 (1.0)	4.4 (5.5)
Journal impact factor		
≤ 2.43	4 (2.0)	6.9 (4.4)
> 2.43	4 (1.0)	3.4 (4.2)
Cancer type		
Genitourinary (n = 1)^a^	4 (NA)	2.6 (NA)
Lower GI (n = 11)	4 (1.0)	3.3 (4)
Lung (n = 8)	4 (0.5)	6.7 (7.6)
Mixed (n = 3)^a^	5 (NA)	4.6 (NA)
Upper GI (n = 13)	4 (1.5)	7.5 (6.7)
Nutrition (n = 21)	4 (2.0)	5.2 (5.4)
Prehabilitation modality		
Exercise (n = 11)	4 (1.0)	5 (5.7)
Multimodal (n = 4)	4 (1.0)	5.9 (7.6)
Risk of bias		
High	4 (1.5)	6.35 (6)
Low/some (n = 16)	4 (1.0)	4.1 (4.5)

*FQ =* $\frac{\text{RFI}}{\text{sample size}} \times 100;$ *IQR* interquartile range

^a^Unable to calculate median and IQR

## Discussion

This systematic review identified 76 trials investigating the effectiveness of prehabilitation on reducing postoperative outcomes of patients undergoing abdominal, pelvic, and thoracic cancer surgery. Within the included trials, a total of 544 postoperative complication outcomes were reported, with 25 outcomes statistically significant, and the other 519 nonsignificant. Overall, the current prehabilitation RCTs are fragile, presenting a median FI of 1 (range 1–13) and a RFI of 4 (range 1–13). Therefore, for most of the prehabilitation RCTs, four patients would be required to have a different response to the treatment for the findings to become statistically significant. Across most subgroups, the median RFI did not change substantially and remained close to 4. When analyzing subgroups within the RCTs that reported postoperative complications as a primary outcome of the study, none of the subgroup analyses identified any large or important differences in FI or RFI. It is also important to compare these findings with those from other surgical areas. For instance, in non-oncologic surgeries, such as orthopedics or bariatrics, fragility indices are similarly low, suggesting that this is a broader challenge in surgical RCTs. This reflects a need for larger, better-powered trials with more consistent outcome reporting.

To the best of the authors’ knowledge, no previous studies have investigated the robustness of RCTs investigating prehabilitation prior to curative cancer surgery. Yet FI has been applied to assess robustness of RCTs for both cancer and surgical populations. In a survey of total neoadjuvant therapy for rectal cancer, the median FI was 2 (IQR 1–16) across 25 outcomes.^1^ This corroborates the finding of our study, where the median FI was 1 across 25 outcomes; both demonstrating highly fragile results. Similarly, when assessing prehabilitation for patients undergoing surgery for colonic diverticular disease, the median FI was 1 across 15 outcomes (range 0–5).^2^ This study by McKechnie et al.^114^ used a FI of 0 to denote nonsignificant outcomes, rather than reporting RFI.^1^ RFI is most often reported in systematic reviews focused on orthopedic surgeries. There is thus no clear comparator for the RFI presented in this study. Our findings align with fragility analyses from non-cancer surgeries. For instance, in colonic diverticular disease, McKechnie et al.^114^ reported a median FI of 1, mirroring our findings. Similarly, in orthopedic and cardiac surgery prehabilitation trials, FI scores are typically low, suggesting a systemic issue in the design and statistical power of prehabilitation RCTs across disciplines.

When calculating FI, it is agreed that there is no singular FI threshold for robustness. Instead, skepticism is encouraged when reviewing RCTs that have a FI that is less than or equal to the number of patients lost to follow-up.^3^ RFI calculation, while equally as valuable as FI calculation, brings slightly more nuance to the evaluation of robustness of RCTs. The RFI score indicates the fragility of a study’s statistical significance. A lower score suggests that small changes in the number of events could alter the study’s conclusions, which raises concerns about the robustness of the findings. Strong scientific evidence should be resilient to small variations in outcomes, providing confidence in the results whether they demonstrate effectiveness or lack of effectiveness of an intervention. Both positive and negative findings contribute valuable knowledge to the field when supported by robust statistical evidence. A RFI or FI score of 1, for instance, still indicates that only one patient would have to have a different response to the treatment for the findings of the RCT to change from nonsignificance to significance, or significance to nonsignificance, respectively. This does not mean that RFI should not be calculated, rather, the opposite is true. RFI gives crucial information that is often lost when only calculating FI. While a high FI will indicate a treatment that is highly likely to yield an outcome, a high RFI indicates a treatment that is highly unlikely to deliver a statistically significant treatment. Thus, for adequate review of the available RCTs for clinical decision-making, RFI should be valued equally as FI. Ultimately, like all statistical measures, RFI and FI should be one of many considerations when determining clinical decisions. Clinically, the implications are substantial. If a study’s outcome can change with only one or two events, the strength of evidence supporting an intervention such as prehabilitation weakens. This is especially crucial when resources are limited and when scaling interventions for broad implementation. Therefore, clinical adoption should be guided not just by statistical significance but by the robustness of that significance.

A limitation of this study is the sole reliance on FI and RFI as an analytical tool. FI and RFI can only be calculated using dichotomous outcomes reported from parallel-arm RCTs.^4^ Thus, continuous outcomes (e.g., serum biomarker levels) cannot be used to assessed for robustness. Continuous outcomes can provide unique information that may add more meaning to postoperative complication risk, and to the efficacy of a trial’s intervention. In addition, trials that have multiple intervention groups being compared against a singular control group are ineligible for FI and RFI analysis. As a result, this limits the number of RCTs that were included in this systematic review. Finally, another key limitation of this study is that while we focus on FI and RFI as statistical measures, these should not be viewed in isolation when making clinical decisions. Statistical significance alone does not necessarily translate to clinical importance or meaningful patient outcomes. Future research should incorporate multiple lines of evidence, including measures of clinical significance, minimal clinically important differences, and patient-reported outcomes, to provide a more comprehensive assessment of treatment effectiveness. This aligns with current movements in clinical research to shift focus beyond pure statistical significance toward more patient-centered and clinically meaningful outcomes. A strength of this study is its scope, which encompasses both RFI and FI. By calculating a study’s RFI, it can be determined whether a study could have been close to statistical significance. Additionally, when calculating RFI and FI through the R fragility package, the group with the smallest number of events is picked. Thus, there are instances in this study where a study will become statistically significant only if the intervention arm produces more postoperative outcomes than the control arm. This should be a focus of future studies, focusing on deeper investigation into calculated RFI and whether the necessary modifications to make a study statistically significant would support a study’s investigation. While our review focused specifically on prehabilitation interventions, future research should consider applying fragility index methodology to outcomes reported in Enhanced Recovery After Surgery (ERAS)-related RCTs. Given the increasing adoption of ERAS protocols, understanding the robustness of their reported benefits would be a valuable complement to our findings.

The currently available RCTs investigating the effectiveness of prehabilitation interventions in patients undergoing cancer surgery are fragile and lack robustness. Clinical decision-making for patients with these cancers should not rest solely on these RCTs. For most trials reporting statistically significant outcomes, only one event needed to be changed for the outcome to lose this significance. For most other included trials where the effect was nonsignificant, four events needed to be changed for an outcome to become statistically significant. The median RFI of the studies indicates that only a small number of event reversals would be needed to change the statistical significance of the findings, reflecting a lack of robustness in the trial evidence, similar to the median FI of included studies.

## Supplementary Information

Below is the link to the electronic supplementary material.

Supplementary file1 (DOCX 25 KB)

Supplementary file2 (DOCX 15 KB)
