# Supplementary material for: How Robust is the Evidence for Prehabilitation in Cancer Surgery?: A Systematic Review and Fragility Index Analysis
Source: Ann Surg Oncol. 2025 Aug 21;33(2):1042–67. doi: 10.1245/s10434-025-18138-3 (PMC12765750; doi:10.1245/s10434-025-18138-3)
Supplement: Supplementary file 1 — Supplementary file1 (DOCX 25 KB) [file 10434_2025_18138_MOESM1_ESM.docx]

| **Supplementary Table 1.** Search strategy and results for Embase, Medline, CENTRAL, AMED, and PsycINFO databases | | | | | | |
| --- | --- | --- | --- | --- | --- | --- |
| **Search term** | | **Embase** | **Medline** | **CENTRAL** | **AMED** | **Psychinfo** |
| 1 | exp randomized controlled trial/ | 798439 | 169449 | 25726 | 3211 | 1528 |
| 2 | randomi?ed controlled trial.mp. | 1085586 | 662788 | 662948 | 5464 | 29962 |
| 3 | exp Controlled Clinical Trial/ | 992758 | 696590 | 38471 | 5249 | 13755 |
| 4 | controlled clinical trial.mp. | 502449 | 114445 | 180060 | 786 | 1851 |
| 5 | randomi?ed.tw. | 1166152 | 811429 | 984098 | 18573 | 108244 |
| 6 | clinical trial.tw. | 293436 | 196200 | 186401 | 3581 | 19030 |
| 7 | trial.tw. | 1148779 | 782670 | 765481 | 14783 | 130339 |
| 8 | quasi-randomi?ed.tw. | 5511 | 4778 | 764 | 103 | 289 |
| 9 | placebo.tw. | 373325 | 250433 | 359849 | 4040 | 44520 |
| 10 | random$.tw. | 2016604 | 1471701 | 1202268 | 26195 | 246204 |
| 11 | RCT.tw. | 55416 | 32849 | 36404 | 644 | 6934 |
| 12 | 1 or 2 or 3 or 4 or 5 or 6 or 7 or 8 or 9 or 10 or 11 | 3002277 | 2100613 | 1478864 | 31989 | 335393 |
| 13 | exp Preoperative Period/ | 424739 | 9446 | 763 | 31 | 336 |
| 14 | pre?operat$.mp. | 630996 | 404827 | 49074 | 2866 | 6439 |
| 15 | before surgery.mp. | 67553 | 46647 | 13359 | 9074 | 1542 |
| 16 | before operation.mp. | 16898 | 9619 | 2843 | 1712 | 115 |
| 17 | exp Preoperative Care/ | 48939 | 72981 | 6719 | 407 | 801 |
| 18 | 13 or 14 or 15 or 16 or 17 | 788855 | 447430 | 60808 | 11696 | 7618 |
| 19 | exp Neoplasms/ | 5833438 | 3906749 | 112689 | 18086 | 62663 |
| 20 | neoplasm$.mp. | 960372 | 3319328 | 108066 | 17452 | 68122 |
| 21 | cancer.mp. | 4619459 | 2246790 | 200918 | 15563 | 74713 |
| 22 | tumo?r$.mp. | 4003377 | 2557948 | 92032 | 6355 | 23670 |
| 23 | malignan$.mp. | 1201200 | 710571 | 32857 | 1749 | 7948 |
| 24 | 19 or 20 or 21 or 22 or 23 | 7494732 | 5275119 | 271936 | 24691 | 104424 |
| 25 | exp Postoperative Complications/ | 844642 | 617587 | 49658 | 1936 | 1303 |
| 26 | post?operat$ complication$.mp. | 466745 | 455386 | 38849 | 1787 | 3068 |
| 27 | adverse effect$.mp. | 310279 | 2165423 | 191210 | 8322 | 20132 |
| 28 | adverse event$.mp. | 459297 | 233618 | 142581 | 1930 | 15197 |
| 29 | Clavien-Dindo.mp. | 14070 | 6591 | 1197 | 1 | 7 |
| 30 | exp Morbidity/ | 450144 | 655642 | 25891 | 240 | 8211 |
| 31 | morbidit$.mp. | 878873 | 503983 | 47864 | 2327 | 32593 |
| 32 | length of hospital stay.mp. | 51410 | 32434 | 8310 | 235 | 1161 |
| 33 | exp "length of stay"/ | 277699 | 103417 | 9874 | 657 | 11322 |
| 34 | length of stay$.mp. | 298302 | 151846 | 24802 | 1414 | 10032 |
| 35 | hospital stay$.mp. | 184868 | 110971 | 26003 | 683 | 4229 |
| 36 | LOS.tw. | 77906 | 68898 | 6271 | 584 | 24156 |
| 37 | 25 or 26 or 27 or 28 or 29 or 30 or 31 or 32 or 33 or 34 or 35 or 36 | 2625150 | 3782540 | 421893 | 15597 | 115653 |
| 38 | exp Exercise/ | 456287 | 251096 | 38616 | 10727 | 32806 |
| 39 | exercis$.mp. | 676268 | 475637 | 138935 | 31304 | 96435 |
| 40 | exp Physical Fitness/ | 44689 | 36708 | 4472 | 2899 | 4992 |
| 41 | physical fitnes$.mp. | 17975 | 36506 | 6557 | 3169 | 8589 |
| 42 | exp Exercise Therapy/ | 103925 | 64459 | 19702 | 9769 | 4336 |
| 43 | exercis$ therap$.mp. | 8440 | 52687 | 16673 | 9796 | 4346 |
| 44 | physical activit$.mp. | 300472 | 158172 | 44190 | 5406 | 51422 |
| 45 | exp Rehabilitation/ | 510142 | 357194 | 50995 | 66611 | 61231 |
| 46 | prehabilitation.mp. | 2445 | 1623 | 655 | 48 | 60 |
| 47 | rehabilit$.mp. | 471712 | 383233 | 66854 | 77220 | 84744 |
| 48 | exercise train$.mp. | 30720 | 22020 | 11103 | 1478 | 1803 |
| 49 | physical therap$.mp. | 49962 | 66564 | 14739 | 14823 | 8255 |
| 50 | 38 or 39 or 40 or 41 or 42 or 43 or 44 or 45 or 46 or 47 or 48 or 49 | 1601121 | 1151476 | 229014 | 105555 | 227735 |
| 51 | exp diet/ | 440088 | 333140 | 25752 | 2123 | 20643 |
| 52 | diet$.mp. | 1243633 | 903848 | 112680 | 8883 | 61269 |
| 53 | nutrition$.mp. | 655510 | 480881 | 54206 | 4830 | 38807 |
| 54 | diet$ counselling.mp. | 1197 | 721 | 525 | 18 | 46 |
| 55 | exp enteric feeding/ | 41144 | 22110 | 2136 | 72 | 621 |
| 56 | enteral nutrition$.mp. | 20198 | 26734 | 5147 | 42 | 542 |
| 57 | exp diet therapy/ | 433743 | 62946 | 7760 | 2229 | 335 |
| 58 | nutrition$ therap$.mp. | 8539 | 7919 | 1314 | 174 | 444 |
| 59 | exp parenteral nutrition/ | 59058 | 25493 | 1917 | 4522 | 17555 |
| 60 | exp nutritional support/ | 23680 | 49471 | 4107 | 1949 | 387 |
| 61 | nutrition$ support.mp. | 32665 | 17653 | 2868 | 112 | 262 |
| 62 | exp food/ | 1345829 | 1491659 | 63840 | 5533 | 1314 |
| 63 | 51 or 52 or 53 or 54 or 55 or 56 or 57 or 58 or 59 or 60 or 61 or 62 | 2704761 | 2329555 | 170524 | 14333 | 101161 |
| 64 | exp cognitive behavioral therapy/ | 28883 | 37289 | 13002 | 1502 | 54911 |
| 65 | cognitive behavio?ral therap$.mp. | 41213 | 39132 | 19379 | 543 | 25216 |
| 66 | Relaxation.mp. | 179690 | 145359 | 15371 | 2791 | 19447 |
| 67 | exp mindfulness/ | 16139 | 6783 | 2172 | 803 | 13041 |
| 68 | mindfulness.mp. | 20833 | 14115 | 9123 | 689 | 21336 |
| 69 | exp coping behavior/ | 85943 | 140714 | 6350 | 4431 | 55568 |
| 70 | coping.mp. | 122329 | 74208 | 11568 | 2797 | 105000 |
| 71 | psychosocial intervention$.mp. | 11566 | 8385 | 2661 | 217 | 7804 |
| 72 | exp psychotherapy/ | 307616 | 220539 | 33358 | 10609 | 224094 |
| 73 | psychotherap$.mp. | 132623 | 101456 | 16694 | 2920 | 217061 |
| 74 | CBT.tw. | 21708 | 14694 | 10381 | 355 | 17927 |
| 75 | exp mental health/ | 246640 | 64186 | 3615 | 2367 | 93987 |
| 76 | Psychological.mp. | 898664 | 679302 | 63876 | 14753 | 589538 |
| 77 | psychoeducation/ | 12188 | 4703 | 4043 | 72 | 5616 |
| 78 | 64 or 65 or 66 or 67 or 68 or 69 or 70 or 71 or 72 or 73 or 74 or 75 or 76 or 77 | 1554257 | 1105260 | 123550 | 29878 | 1006139 |
| 79 | 50 or 63 or 78 | 5391573 | 4311922 | 452546 | 137854 | 1252265 |
| 80 | 12 and 18 and 24 and 37 and 79 | 1663 | 954 | 1191 | 27 | 8 |
