# Supplementary material for: How Robust is the Evidence for Prehabilitation in Cancer Surgery?: A Systematic Review and Fragility Index Analysis
Source: Ann Surg Oncol. 2025 Aug 21;33(2):1042–67. doi: 10.1245/s10434-025-18138-3 (PMC12765750; doi:10.1245/s10434-025-18138-3)
Supplement: Supplementary file 2 — Supplementary file2 (DOCX 15 KB) [file 10434_2025_18138_MOESM2_ESM.docx]

| **Supplementary Table 2.** Search strategy and results for CINAHL database | | |
| --- | --- | --- |
| **Search term** | | **CINAHL** |
| S1 | (MH "Randomized Controlled Trials+") OR (MH "Clinical Trials+") | 354485 |
| S2 | randomized controlled trials OR randomized control trial OR rtc OR randomized control trials OR randomized controlled trial OR clinical trial OR TI trial OR TI random OR controlled trial | 512849 |
| S3 | S1 OR S2 | 538244 |
| S4 | (MH "Preoperative Care+") | 25496 |
| S5 | preoperative OR preoperative care OR preop* OR pre-op* OR pre-operative OR pre-op OR perioperative OR peri operative OR before surgery | 153575 |
| S6 | S4 OR S5 | 157382 |
| S7 | (MH "Cancer Patients") | 51171 |
| S8 | (MH "Neoplasms, Squamous Cell+") | 25738 |
| S9 | cancer patients OR oncology patients OR patients with cancer OR cancer OR neoplasms OR oncology OR cancers OR tumour OR tumor OR malignancy | 863562 |
| S10 | S7 OR S8 OR S9 | 864714 |
| S11 | postoperative complications OR postoperative issues OR postoperative problems OR surgical complications OR adverse effects OR RP adverse events OR morbidity OR length of stay OR los OR inpatient stay OR time in hospital OR time to discharge | 825015 |
| S12 | hospitalization OR hospital stay OR length of stay OR stay length OR hospitalisation OR hospital stay | 176126 |
| S13 | S11 OR S12 | 904843 |
| S14 | (MH "Enteral Nutrition") OR (MH "Parenteral Nutrition Solutions") | 11523 |
| S15 | (MH "Exercise+") | 130212 |
| S16 | exercise OR physical activity OR fitness OR physical therapy OR rehabilitation OR prehabilitation OR prehab OR pre-operative rehabilitation OR peri-operative rehabilitation OR exercise training OR physical activity OR physical exercise | 514993 |
| S17 | diet OR nutrition OR food habit OR eating habit OR lifestyle OR food OR dietary supplements OR nutritional support OR nutrition OR enteral feeding OR tube feeding OR enter* | 556496 |
| S18 | (MH "Mental Health") | 59336 |
| S19 | (MH "Cognitive Therapy+") | 30090 |
| S20 | (MH "Rehabilitation, Psychosocial+") OR (MH "Psychosocial Intervention") | 6633 |
| S21 | (MH "Behavior Therapy+") | 43019 |
| S22 | (MH "Health Food+") | 8281 |
| S23 | (MH "Psychology, Educational+") | 71257 |
| S24 | (MH "Psychology, Clinical+") | 1289 |
| S25 | (MH "Psychological Well-Being") | 38711 |
| S26 | (MH "Psychotherapy, Group+") | 26375 |
| S27 | food OR cognitive behavioral therapy OR relaxation OR coping OR psychology OR psychotherapy OR psychological OR psychosocial OR mindfulness OR psychoeducation OR mental health | 1234441 |
| S28 | S14 OR S15 OR S16 OR S17 OR S18 OR S19 OR S20 OR S21 OR S22 OR S23 OR S24 OR S25 OR S26 OR S27 | 1965661 |
| S29 | S3 AND S6 AND S10 AND S13 AND S28 | 351 |
